# Symptoms of Raynaud’s phenomenon (RP) in fibromyalgia syndrome are similar to those reported in primary RP despite differences in objective assessment of digital microvascular function and morphology

**DOI:** 10.1007/s00296-016-3483-6

**Published:** 2016-05-02

**Authors:** M. Scolnik, B. Vasta, D. J. Hart, J. A. Shipley, N. J. McHugh, J. D. Pauling

**Affiliations:** 1 Department of Rheumatology, Royal National Hospital for Rheumatic Diseases, Upper Borough Walls, Bath, BA1 1RL UK; 2 Sección Reumatología, Hospital Italiano de Buenos Aires, Buenos Aires, Argentina; 3 Department of Pharmacy and Pharmacology, University of Bath, Bath, UK

**Keywords:** Raynaud’s phenomenon, Fibromyalgia syndrome, Thermography, Objective, Self-report, Classification

## Abstract

Symptoms of Raynaud’s phenomenon (RP) are common in fibromyalgia syndrome (FMS). We compared symptom characteristics and objective assessment of digital microvascular function using infrared thermography (and nailfold capillaroscopy where available) in patients with FMS (reporting RP symptoms) and primary RP. We retrospectively reviewed the outcome of microvascular imaging studies and RP symptom characteristics (captured using patient-completed questionnaire at the time of assessment) for patients with FMS (reporting RP symptoms) and patients with primary RP referred for thermographic assessment of RP symptoms over a 2-year period. Of 257 patients referred for thermographic assessment of RP symptoms between 2010 and 2012, we identified 85 patients with primary RP and 43 patients with FMS. There were no differences in RP symptom characteristics between FMS and primary RP (*p* > 0.05 for all comparisons). In contrast, patients with FMS had higher baseline temperature of the digits (32.1 vs. 29.0 °C, *p* = 0.004), dorsum (31.9 vs. 30.2 °C, *p* = 0.005) and thermal gradient (temperature of digits minus temperature of dorsum; +0.0 vs. −0.9 °C, *p* = 0.03) compared with primary RP. Significant differences between groups persisted following local cold challenge. In primary RP, patient reporting “blue” digits, bi-phasic and tri-phasic RP was associated with lower digital perfusion. In contrast, no associations between skin temperature and RP digital colour changes/phases were identified in FMS. Our findings suggest that symptoms of RP in FMS may have a different aetiology to those seen in primary RP. These findings have potential implications for both the classification of RP symptoms and the management of RP symptoms in the context of FMS. Digital colour changes reported by patients might reflect the degree of digital microvascular compromise in primary RP.

## Introduction

Raynaud’s phenomenon (RP) describes intermittent excessive vasoconstriction of digital vessels in response to cold exposure and emotional stress [1]. Attacks of RP are typically associated with digital cutaneous colour changes including a “white” phase (vasoconstriction), a “blue” phase (cyanosis) and a “red” phase (reactive hyperaemia) [1]. Due to its episodic nature, objective evidence of digital microvascular dysfunction is not typically evident on clinical assessment, which has led to reliance upon patient self-report. A history of increased sensitivity to cold and the presence of two digital colour changes are considered sufficient to make a “definite” diagnosis of RP [2]. Recent studies have reported a high frequency of RP symptoms (18.2–53.3 %) in patients with fibromyalgia syndrome (FMS) [3–6]. Structural and functional microvascular disturbance has been reported in FMS compared with healthy controls [6–9]. To date, no studies have compared and contrasted RP symptom characteristics and outcome of microvascular imaging studies in FMS (reporting RP symptoms) and primary RP. We retrospectively assessed RP symptom characteristics and microvascular imaging study findings (infrared thermography ± nailfold capillaroscopy [NC]) in patients with a clinician diagnosis of either FMS or primary RP referred for assessment of RP symptoms over a 2-year period.

## Materials and methods

A retrospective review of patients referred for thermographic evaluation of RP symptoms at our unit between 2010 and 2012 was undertaken. Local research and development approval was obtained for the study (Ref RBB389). We received written confirmation from the National Research Ethics Service confirming that Research Ethics Committee approval was not required for a retrospective review of data obtained under normal clinical practice [under UK-wide Governance Arrangements for Research Ethics Committees (GAfREC)].

### Subjects

A retrospective case note review of patients referred for thermographic evaluation of RP symptoms was undertaken to determine diagnosis. A diagnosis of primary RP was based on a final clinician diagnosis of “primary RP”, but also required the absence of clinical features of an underlying rheumatic disease or CTD and a negative ANA (immunofluorescence on Hep-2 cells negative at >1:160 dilution). A diagnosis of FMS required was based on documentation of widespread pain and tenderness of at least 11 of the 18 FMS trigger points using 1990 ACR criteria [11] and/or a final clinician diagnosis of FMS.

### Patient questionnaire

All patients referred for thermographic assessment of RP symptoms complete a short questionnaire during 20-min acclimatization at 23 °C. The questionnaire captures clinical characteristics of RP using criteria derived from the consensus view of the UK Scleroderma Study Group in an earlier validation study [2]. The questionnaire records whether fingers are sensitive to cold, whether they show digital colour changes (white, red, blue and purple), whether patients experience numbness or paraesthesia in response to cold and the presence of symptoms in the absence of cold exposure. The questionnaire also collects relevant information including smoking history, cardiovascular risk factors (diabetes mellitus, dyslipidaemia and hypertension) and vasodilator medication use to aid subsequent interpretation of microvascular imaging studies.

### Thermal imaging protocol and analysis

All patients had undergone a cold stress test (CST) under the same standardized conditions. Following 20-min acclimatization (at 23 ± 0.5 °C), thermographic images were taken of the dorsum of the hands (Thermovision Camera, FLIR systems, Danderyd, Sweden). Patients then submerged their hands (in polythene gloves to avoid evaporative cooling) to the level of the radio-carpal joint into a water bath cooled to 20 °C (±0.1 °C) for 60 s. A second thermal image of the dorsum of the hands was captured 10 min following cold challenge. Images were processed using commercially available software (CTHERM, version 2.3, University of Glamorgan). Regions of interest encompassing the 2nd–5th digits (distal to metacarpophalangeal joints) and dorsum of hand were prepared and the mean surface skin temperature within each region of interest recorded. The thermal gradient (TG) was calculated by subtracting the mean temperature of the dorsum of the hands from the mean temperature of the digits. A mean thermal gradient of both hands was calculated at both baseline and following cold challenge. In healthy controls, the TG is typically positive (~+1 to 2 °C) as the digital temperature (~33 °C) is typically greater than the dorsum of the hand (~31 °C) at baseline assessment and following cold challenge due to the action of thermoregulatory arteriovenous anastomoses (AVAs) within glabrous regions of the fingertips. In Raynaud’s phenomenon, the TG is typically negative (−1 to 3 °C) due to a lower digital temperature (~26–28 °C) in comparison with the dorsum of the hand (~31 °C) due to closure of thermoregulatory AVAs in resting state or in response to cold provocation. The TG (and related thermographic parameters such as the distal dorsal difference) has been successfully applied to differentiate disease states in populations of healthy controls, primary RP and secondary RP.

### Nailfold capillaroscopy protocol and analysis

NC (when requested at the discretion of the treating clinicians) was performed at the index, middle and ring fingers of each hand using a pillar-mounted 1.3 megapixel camera (PL-A742, PixeLINK) at a magnification of 200×. Images were analysed using PixeLINK Capture OEM software. NC images of all digits were reviewed (without knowledge of diagnosis) by two blinded assessors (BV and MS), and patients were categorized as having either normal, mild non-specific abnormalities or scleroderma-pattern abnormalities using classification criteria proposed by Cutolo et al. [10]. Indeterminate cases were reviewed by a blinded third assessor (JP) and consensus reached.

### Statistical analysis

Comparisons were made between the two patient groups for baseline demographics (age, smoking history, vasodilator medication use and cardiovascular disease), RP symptom characteristics, thermographic analysis at baseline and following CST (absolute skin temperature at digits, dorsum and thermal gradient) and NC changes (when available). Additional analyses were undertaken to evaluate potential associations between the type and number of digital colour changes reported by patients and the outcome of thermographic assessments. Categorical variables are reported as numbers and/or percentages and were compared using Chi-square test. Quantitative variables are reported as medians (interquartile range), and differences were assessed using Mann–Whitney *U* test. All analyses were two-tailed, and a *p* value of ≤0.05 was considered statistically significant.

## Results

Two hundred and fifty-seven patients underwent thermographic assessment of RP symptoms between 2010 and 2012. The case note review identified 128 patients who fulfilled our criteria for either primary RP (*n* = 85) or FMS (*n* = 43). NC had been undertaken in 66 patients (48 primary RP). There was a greater number of current smokers in the FMS group (37.2 vs. 16.5 %, *p* = 0.009) but no other statistically significant differences between groups in terms of baseline demographics, conventional cardiovascular risk factors or vasodilator use (Table 1). No differences were identified between primary FMS and primary RP for either individual reported digital colour changes, the number of digital colour changes (i.e. mono-phasic, bi-phasic, etc.) or other symptom characteristics (Table 1). For each group, “white” was the most commonly reported digital colour change (~68 %) with similar proportions of patients (~30 %) reporting mono-phasic, bi-phasic and tri-phasic RP attacks.

**Table 1:** Baseline characteristics, questionnaire results, thermographic assessment and nailfold capillaroscopy in primary RP and FMS

	Primary RP (*n* = 85)	FMS (*n* = 43)	*p* value
Age, mean (SD)	47.8 (14.7)	43.8 (10.8)	0.12
Female, *n* (%)	66 (77.6)	39 (90.7)	0.07
Smoking history, *n* (%)			
Never	40 (47)	19 (44.2)	0.76
Ex	31 (36.5)	8 (18.6)	0.04
Current	14 (16.5)	16 (37.2)	0.009
Diabetes mellitus, *n* (%)	2 (2.4)	2 (4.7)	0.48
Hypertension, *n* (%)	16 (18.8)	3 (7.0)	0.08
Hyperlipidaemia, *n* (%)	11 (12.9)	1 (2.3)	0.05
Vasodilator use, *n* (%)	21 (24.7)	14 (32.6)	0.35
*Questionnaire results*			
Cold intolerance, *n* (%)	80 (94.1)	42 (97.7)	0.37
White, *n* (%)	60 (69.9)	29 (67.4)	0.71
Blue, *n* (%)	38 (44.7)	14 (32.6)	0.19
Red, *n* (%)	37 (43.5)	21 (48.8)	0.57
Purple, *n* (%)	41 (48.2)	16 (37.2)	0.24
Mono-phasic, *n* (%)	23 (27.1)	14 (32.6)	0.52
Bi-phasic, *n* (%)	21 (24.7)	11 (25.6)	0.91
Tri-phasic, *n* (%)	32 (37.6)	13 (30.2)	0.41
Quadriphasic, *n* (%)	9 (10.6)	5 (11.6)	0.86
Numbness, *n* (%)	73 (85.9)	41 (95.3)	0.11
Symptoms in absence of cold, *n* (%)	47 (55.3)	25 (58.1)	0.76
Thumbs involvement, *n* (%)	31 (36.5)	17 (39.5)	0.74
Toes involvement, *n* (%)	66 (77.6)	35 (81.4)	0.62
*Thermal imaging*			
Baseline temperature digits, °C (IQR)	29.0 (7.3)	32.1 (7.3)	0.004
Baseline temperature dorsum, °C (IQR)	30.2 (4.4)	31.9 (4.3)	0.005
Baseline thermal gradient, °C (IQR)	−0.9 (3.1)	0.0 (3.0)	0.033
Post-CST temperature digits, °C (IQR)	26.5 (7.4)	30.8 (8.6)	0.003
Post-CST temperature dorsum, °C (IQR)	28.8 (4.1)	30.7 (4.6)	0.003
Post-CST thermal gradient, °C (IQR)	−2.6 (4.1)	−0.2 (3.6)	0.066

	Primary RP (*n* = 48)	FMS (*n* = 18)	*p* value
*Nailfold capillaroscopy*			
Normal nailfold capillaroscopy, *n* (%)	31 (64.6)	15 (83.3)	0.14
Subtle non-specific NC changes, *n* (%)	15 (31.3)	3 (16.7)	0.24
Scleroderma-like NC changes, *n* (%)	2 (4.1)	0 (0)	0.38

Analyses of categorical data used Chi-square test. Analysis of continuous data used Mann–Whitney *U* test

*RP* Raynaud’s phenomenon, *FMS* fibromyalgia syndrome, *IQR* interquartile range, *CST* cold stress test, *NC* nailfold capillaroscopy

In contrast, significantly lower skin temperature of the digits [29.0 (7.3) vs. 32.1 °C (7.3), *p* = 0.004], dorsum [30.2 (4.4) vs. 31.9 °C (4.3), *p* = 0.005] and thermal gradient [−0.9 (3.1) vs. +0.0 °C (3.0), *p* = 0.03] was identified in primary RP compared with FMS. Following CST, patients with primary RP continued to have significantly lower skin temperature at the digits and dorsum of the hand, with a strong trend for a lower TG post-CST compared with FMS (Table 1).

The majority of patients with both primary RP and FMS had normal NC (64.6 and 83.3 %, respectively). Subtle NC changes were found in primary RP more commonly than in FMS although this trend did not achieve statistical significance (31.3 vs. 16.7 %, *p* = 0.24). Due to the blinded nature of our NC review, two patients with primary RP (4.1 %) were identified in this study as having “early” scleroderma NC changes. Both patients were ANA negative and did not have any clinical features of SSc (or alternative CTD). Repeat statistical analysis excluding these two subjects from the primary RP group did not influence the outcome of the study (data not reported). There were no significant differences in skin temperature of the digits, dorsum or thermal gradient in patients with primary RP with normal NC versus abnormal NC changes (*p* > 0.05 for all comparisons). In contrast, FMS patients with subtle NC changes compared with those with normal NC appearances appeared to have slightly lower skin temperature of the digits (26.0 vs. 32.8 °C, *p* = 0.09) and a significantly lower thermal gradient (−2.4 vs. +0.5 °C, *p* = 0.006) although these findings must be considered with caution in the context of a low number of patients with FMS and NC changes (*n* = 3).

We examined the possible relationship between individuals reported digital colour changes and thermographic assessment. Across the whole cohort, reporting “blue” digital colour changes was associated with a significantly lower baseline digital (27.8 vs. 31.7 °C, *p* = 0.005) and baseline dorsal (29.7 vs. 31.4 °C, *p* = 0.007) skin temperature, along with a corresponding lower TG (−1.8 vs. −0.2 °C, *p* = 0.06). Significant associations were also identified in patients reporting “purple” colour changes (Table 2). When assessing the primary RP group in isolation, significantly lower baseline skin temperature was identified in patients reporting “blue” digital colour changes for the digits (27.4 vs. 30.5 °C, *p* = 0.017) and dorsum (29.5 vs. 31.1 °C, *p* = 0.023) of the hands (Table 2). Similar associations between “blue” and “purple” reported digital colour changes and skin temperature (of the digits and dorsum) were identified following cold challenge (data not reported). In contrast, there was no association between reported digital colour changes and thermographic assessment of the hands at baseline (or following cold challenge) in patients with FMS (Table 2).

**Table 2:** Relationship between reported colour changes and baseline thermographic assessment

Colour change	Thermographic assessment	Symptom absent	Symptom present	*p* value
*Whole cohort*				
White	Digits	31 (8.3)	29.2 (7.5)	0.59
	Dorsum	31.1 (4.8)	30.4 (4.2)	0.57
	Thermal gradient	−0.4 (3.1)	−0.6 (3.0)	0.36
Blue	Digits	31.7 (7.4)	27.8 (7.0)	0.005
	Dorsum	31.4 (4.3)	29.7 (4.7)	0.007
	Thermal gradient	−0.20 (2.6)	−1.8 (3.2)	0.059
Red	Digits	30.1 (7.4)	30 (7.8)	0.87
	Dorsum	30.4 (4.2)	30.7 (4.7)	0.88
	Thermal gradient	−0.5 (3.2)	−0.7 (2.8)	0.64
Purple	Digits	31.4 (6.6)	28.5 (8.0)	0.031
	Dorsum	31.3 (3.8)	29.8 (4.9)	0.05
	Thermal gradient	−0.3 (2.5)	−1.1 (3.4)	0.067
*Primary Raynaud’s phenomenon*				
White	Digits	31.4 (8.3)	27.9 (7.2)	0.20
	Dorsum	31.6 (4.7)	29.8 (4.6)	0.12
	Thermal gradient	−0.4 (3.4)	−1.5 (3.0)	0.38
Blue	Digits	30.5 (7.7)	27.4 (8.6)	0.017
	Dorsum	31.1 (4.3)	29.5 (5.0)	0.023
	Thermal gradient	−0.4 (2.8)	−2.0 (3.2)	0.133
Red	Digits	29.5 (7.4)	27.4 (7.3)	0.52
	Dorsum	30.3 (4.3)	29.6 (4.8)	0.48
	Thermal gradient	−0.6 (3.3)	−1.4 (2.8)	0.61
Purple	Digits	30.4 (6.8)	27.2 (8.0)	0.13
	Dorsum	30.4 (4.0)	29.6 (5.5)	0.20
	Thermal gradient	−0.4 (2.4)	−1.8 (3.3)	0.12
*Fibromyalgia syndrome*				
White	Digits	30.8 (8.2)	32.2 (7.4)	0.30
	Dorsum	30.6 (5.0)	32.0 (4.2)	0.15
	Thermal gradient	0.1	−0.01 (3.3)	0.60
Blue	Digits	32.5 (6.4)	30.8 (7.2)	0.22
	Dorsum	32.1 (3.8)	31.4 (4.2)	0.31
	Thermal gradient	0.02 (2.5)	−0.7 (3.7)	0.48
Red	Digits	31.4 (7.1)	32.2 (7.5)	0.94
	Dorsum	31.4 (4.1)	32.0 (4.3)	0.68
	Thermal gradient	0.2 (3.2)	−0.02 (2.9)	0.42
Purple	Digits	32.3 (6.0)	31.2 (7.9)	0.09
	Dorsum	32.1 (3.6)	30.8 (4.1)	0.15
	Thermal gradient	0.02 (2.9)	−0.01 (3.8)	0.35

Exploring the relationship between the number of colour changes in RP attacks and thermographic assessment identified some intriguing observations. Bi- and tri-phasic RP was associated with lower baseline digital and dorsum skin temperature compared with mono-phasic RP (Table 3). Digital and dorsal skin temperatures remained significantly lower in tri-phasic compared with mono-phasic RP following cold challenge in primary RP. In contrast, bi- and tri-phasic RP was associated with higher baseline skin temperature compared with mono-phasic RP in FMS (Table 3). No significant associations between the number of colour changes reported and thermographic assessment following cold challenge were identified in FMS.

**Table 3:** Relationship between number of digital colour changes and baseline thermographic assessment

Diagnosis	Thermographic assessment	Number of digital colour changes reported			
		1	2	3	4
Primary RP	Digits	31.7 (4.1)	27.3 (7.4)^†^	27.2 (7.7)**	29 (8.7)
	Dorsum	31.9 (2.3)	29.6 (8.8)*	29.3 (5.3)**	29.4 (4.7)
	Thermal gradient	−0.3 (1.6)	−2.0 (3.5)	−1.8 (3.4)*	−0.4 (3.9)
FMS	Digits	30.1 (7.8)	33.0 (3.3)^§^	31.8 (7.3)	31.9 (6.9)
	Dorsum	31.6 (5.3)	32.8 (3.6)	31.6 (4.0)	31.1 (4.7)
	Thermal gradient	−0.4 (3.5)	0.5 (0.7)^§§^	−0.4 (3.1)	0.0 (2.7)

*RP* Raynaud’s phenomenon, *FMS* fibromyalgia syndrome, *TG* thermal gradient

* *p* < 0.05 versus one colour change; ** *p* = 0.004 versus one colour change; ^†^ *p* = 0.06 versus one; ^§^ *p* < 0.05 versus three colour changes; ^§§^ *p* = 0.006 versus three colour changes

## Discussion

The present study has identified similar patient-reported characteristics of RP in patients with FMS (reporting RP symptoms) compared with primary RP, despite little objective evidence of digital microvascular compromise in FMS on objective imaging. Our findings would not support an association between FMS and digital microvascular dysfunction and suggest that RP symptoms in FMS may be caused by different mechanisms than those seen in primary RP or secondary to the autoimmune conditions like SSc.

Our findings were in contrast with earlier smaller studies, suggesting evidence of microcirculatory dysfunction in FMS [6–9, 12]. The present study benefits from being comparatively much larger than previous work and from having evaluated an enriched cohort of FMS patients specifically referred for objective assessment of RP symptoms. A prospective cross-sectional study of unselected patients with FMS would be the preferred method for identifying the true burden of RP symptoms in FMS.

The chief limitation of the study was its retrospective design, and additional prospective studies are required to verify these findings. We relied heavily upon clinician diagnosis for disease classification, but this did include assessments such as ANA and trigger point assessments that form part of existing classification criteria for primary RP and FMS. It might be argued that the inclusion of patients reporting mono-phasic RP and subtle NC changes is not compatible with existing classification criteria for primary RP [2, 13]. The lower skin temperatures identified in primary RP patients reporting bi-phasic and tri-phasic RP suggests some merit in requiring at least two digital colour changes to diagnose RP as has been proposed in classification criteria for both RP and systemic sclerosis (SSc) [2, 14]. In contrast, a higher number of reported digital colour changes were associated with higher skin temperatures in FMS highlighting a pitfall in this approach. A strength of the study was the efforts made to blind assessors to the clinical diagnosis when analysing microvascular imaging outcomes. Patients with NC changes generally had subtle changes that fell short of the NC classification criteria for SSc microangiopathy defined by Cutolo et al. [10]. Most treating clinicians had regarded the NC appearances as within normal limits when considering the images in the context of their clinical and serological findings (hence the diagnosis of primary RP). Subtle NC changes have been identified previously in primary RP, and it has been suggested that primary RP may represent a *form fruste* of scleroderma-spectrum disease [15–17].

This study has implications for existing classification criteria of RP-related disorders which heavily rely on patient self-report of digital colour changes [2, 13, 14, 18]. Digital vasoconstriction in response to cold exposure is an important component of healthy thermoregulation, leading one early twentieth-century physician to observe “we are all subjects of Raynaud’s phenomena to a greater or lesser degree” [19]. Our findings suggest that symptoms of RP in FMS may have a different aetiology than those seen in primary RP, which might have implications for the treatment of RP symptoms in the context of FMS, where the safety and efficacy profiles of vasodilator therapy might differ to that demonstrated in previous studies of primary RP.

## Abbreviations

RP: Raynaud’s phenomenon
FMS: Fibromyalgia syndrome
NC: Nailfold capillaroscopy
TG: Thermal gradient
AVAs: Arteriovenous anastamoses
SSc: Systemic sclerosis
